# SUBJECTIVE FINANCIAL STRAIN AMONG CAREGIVERS OF INDIVIDUALS WITH ALZHEIMER’S DISEASE AND DEMENTIA

**DOI:** 10.1093/geroni/igae098.3895

**Published:** 2024-12-31

**Authors:** Yujun Zhu, Susan Enguidanos, Kathleen Wilber, Donna Benton, Francesca Falzarano

**Affiliations:** University of Southern California, Los Angeles, California, United States; University of Southern California, Los Angeles, California, United States; University of Southern California, Los Angeles, California, United States; University of Southern California, Los Angeles, California, United States; University of Southern California, Los Angeles, California, United States

## Abstract

Caregivers of individuals with Alzheimer’s disease and dementia often face significant caregiving burdens. While the cost of care is admittedly high for dementia patients, research on the financial strain experienced by family caregivers remains limited. This cross-sectional study used publicly available data from the “Caregiving in the U.S. 2020” survey of 1,241 caregivers who identified as caring for an adult age 50 and older. Comparative analyses were conducted to assess subjective financial strain and caregiving intensity among caregivers of individuals with and without Alzheimer’s disease/dementia. Logistic regression models were used to explore the association between caring for individuals with Alzheimer’s disease/dementia and family caregivers’ subjective financial strain after controlling for caregivers’ demographic characteristics, care recipients’ conditions and care intensity. The average age of caregivers was 56.1 (SD=16.5) years and 59% were female. About 61% of caregivers for individuals with Alzheimer’s disease/dementia reported financial strain, significantly higher than the 51% reported by caregivers of individuals without these conditions (P<.001). Caregivers assisted in more activities of daily living (2.1 vs. 1.4) and instrumental activities of daily living (4.9 vs. 4.3) in the Alzheimer’s group compared to the non-Alzheimer’s caregivers. Caring for recipients with Alzheimer’s disease/dementia was associated with a higher (OR=1.5, 95% CI: 1.1-2.0) likelihood of experiencing subjective financial strain. This study highlights the amplified financial challenges faced by caregivers of individuals with Alzheimer’s disease and dementia. The results underscore the necessity for comprehensive support systems, including financial assistance programs to alleviate the burdens on this population.

